# Supplementary material for: The Association between Plasma Concentration of Phytoestrogens and Hypertension within the Korean Multicenter Cancer Cohort
Source: Nutrients. 2021 Dec 5;13(12):4366. doi: 10.3390/nu13124366 (PMC8703377; doi:10.3390/nu13124366)
Supplement: Supplementary file 1 [file nutrients-13-04366-s001.zip › nutrients-1445365-supplementary.pdf]

Supplementary Table S1. Partial regression coefficients controlled for confounding factors between phytoestrogens and prehypertension/hypertension.

	Genistein		Daidzein		Equol		Enterolactone	
	**Prehypertension**	**Hypertension**	**Prehypertension**	**Hypertension**	**Prehypertension**	**Hypertension**	**Prehypertension**	**Hypertension**
	Partial regression coefficient		Partial regression coefficient		Partial regression coefficient		Partial regression coefficient	
Age	0.02	0.06***	0.02	0.06***	0.01	0.06***	0.02	0.07***
Sex (Female)	0.12	0.02	0.19	0.05	0.16	-0.02	0.25	0.06
Enrolment year	-0.19***	-0.08	-0.20***	-0.09	-0.19***	-0.09	-0.19***	-0.08
Education	-0.10	0.29	-0.14	0.29	-0.14	0.27	-0.05	0.37
Smoker	0.30	-0.24	0.39	-0.17	0.34	-0.29	0.35	-0.30
Drinker	0.85*	0.66*	0.91**	0.67*	0.90**	0.65*	0.79*	0.52
Obesity	0.06	0.14***	0.07	0.14***	0.07	0.14***	0.06	0.13***
Isoflavones								
Genistein								
1T (< 101)	1.00	1.00						
2T (101-296.4)	0.64	0.08						
3T (≥ 296.5)	-0.38	-0.11						
Hosmer and Lemeshow test	0.25	0.10						
Daidzein								
1T (< 57.5)			1.00	1.00				
2T (57.5-208.9)			-0.28	-0.55				
3T (≥ 209)			-0.50	-0.13				
Hosmer and Lemeshow test			0.08	0.40				
Equol								
1T (< 20.5)					1.00	1.00		
2T (20.5-58.9)					-0.43	-0.47		
3T (≥ 59)					-0.68**	-0.85***		
Hosmer and Lemeshow test					0.30	0.97		
Lignans								
Enterolactone								
1T (< 31.6)							1.00	1.00
2T (31.6-76.35)							-0.40	-0.40
3T (≥ 76.4)							-0.96***	-1.29***
Hosmer and Lemeshow test							0.78	0.26

*P < 0.05; ** P<0.01; ***P < 0.001.
